# Spatial Organization in Self-Initiated Visual Working Memory

**DOI:** 10.3389/fpsyg.2019.02734

**Published:** 2019-12-13

**Authors:** Hagit Magen, Tatiana Aloi Emmanouil

**Affiliations:** ^1^School of Occupational Therapy, The Hebrew University, Jerusalem, Israel; ^2^Program in Psychology, Psychology Department, The Graduate Center, Baruch College, City University of New York, New York, NY, United States

**Keywords:** visual working memory, self-initiation, Gestalt, grouping, metacognition, metamemory

## Abstract

Ample research in visual working memory (VWM) has demonstrated that the memorized items are maintained in integrated spatial configurations, even when the spatial context is task irrelevant. These insights were obtained in studies in which participants were provided with the information they memorized. However, the encoding of provided information is only one aspect of memory. In everyday life, individuals often construct their own memory representations, an aspect of memory we have previously termed self-initiated (SI) working memory. In this study, we employed a SI VWM task in which participants selected the visual targets they memorized. The spatial locations of the targets were task irrelevant. Nevertheless, we were interested to see whether participants would construct spatially structured memory representations, which would suggest that they intended to maintain the visual targets as integrated spatial configurations. The results of two experiments demonstrated that participants constructed spatially structured configurations relative to random displays. Specifically, participants selected visual targets in close spatial proximity and constructed spatial sequences with short distances and fewer path crossings. When asked to construct configurations for a hypothetical competitor in a memory contest, participants disrupted the spatial structure by selecting visual targets that were further apart and by increasing the distances between them, which suggests that these characteristics were under their control. At the end of each experiment, participants provided verbal descriptions of the strategies they used to construct the memory displays. While the spatial structure of the SI memory representations was robust, it was absent from the participants’ explicit descriptions, which focused on non-spatial strategies. Participants reported selecting items based, most frequently, on semantic categories and visual features. Taken together, these results demonstrated that participants had access to the metacognitive knowledge on the spatial structure of VWM representations, knowledge they manipulated to construct memory representations that enhanced or disrupted memory performance. While having a profound impact on behavior, this metacognitive knowledge on spatial structure remained implicit, as it was absent from the participants’ verbal reports. Viewed from a larger perspective, this study explores how individuals interact with the world by actively structuring their surroundings to maximize cognitive performance.

Our everyday interaction with a visually rich and complex world is often aided by short-lived internal representations of relevant information from our surroundings. Visual working memory (VWM) is the mechanism in charge of the formation and temporary maintenance of such representations (see Luck and Vogel, 2013; Ma et al., 2014, for reviews). While rich and complex, the world is also highly structured and governed by spatial regularities such as those captured by the Gestalt organization cues (Wagemans et al., 2012). Consistent with the complexity and inherent structure in our surroundings, the basic representations of VWM are also complex, consisting of interconnected multi-level visual objects (Jiang et al., 2000; Brady et al., 2011; Orhan and Jacobs, 2014). Moreover, memory representations that follow real world regularities typically benefit VWM performance. For instance, visual displays in which items are grouped by any number of Gestalt organization cues such as proximity, similarity, connectedness or symmetry, yield higher accuracy rates relative to unstructured displays (e.g., Woodman et al., 2003; Rossi-Arnaud et al., 2006; Peterson and Berryhill, 2013; Gao et al., 2015; Jiang et al., 2016; van Lamsweerde et al., 2016). Structured displays appear to benefit performance by reducing cognitive and neural loads due to the compression of maintained information into higher order configurations (i.e., chunks, Miller, 1956), that effectively increases memory capacity (Xu and Chun, 2007; Gao et al., 2011; Luria and Vogel, 2014; Peterson et al., 2015).

Spatially structured memory displays, such as those based on Gestalt cues, encourage grouping and consequently the maintenance of independent visual items as integrated spatial configurations. However, other lines of research suggest that the formation and maintenance of spatially integrated memory representations is more fundamental to VWM and occurs even when the encoded visual displays are unstructured and space is overall task irrelevant (Gratton, 1998; Jiang et al., 2000; Treisman and Zhang, 2006). For instance, in a color VWM task in which participants were probed on individual colored items, Jiang et al. (2000) changed the irrelevant locations of the memory targets between encoding and retrieval (i.e., disrupted the overall spatial configuration of the encoded display during retrieval). The disruption of the spatial configuration during the retrieval phase decreased memory performance, suggesting that individual items were encoded and maintained as integrated spatial configurations, even when the displays were spatially unstructured. Thus, space seems to have a unique and fundamental role in VWM.

In the studies reviewed thus far, participants memorized visual displays that were provided to them, and therefore had no control over the memorized content. From these displays, participants extracted and subsequently maintained the overall spatial configuration of the display, a process that is thought to occur quickly and relatively effortlessly (Jiang et al., 2000). The maintenance of provided information, however, is only one aspect of memory performance in everyday life. In many scenarios, memory is self-initiated as individuals shape the content of their own memory representations. For example, individuals often place objects in different locations and retrieve them a short while after. We have recently begun to explore this aspect of memory, we termed self-initiated (SI) WM, which although is prevalent in everyday behavior, is largely unexplored in the WM literature (Magen and Emmanouil, 2018, 2019; Magen and Berger-Mandelbaum, 2018; Milchgrub and Magen, 2018; Berger-Mandelbaum and Magen, 2019).

An important question regarding SI WM is whether individuals construct memory representations with proporties that are consistent with the basic function and structure of memory. Put differently, assuming that individuals select memory representations with an attempt to maximize performance, would they have access to the metacognitive knowledge of the structure of efficient WM representations, knowledge that would allow SI WM to operate efficiently. Given the fundamental role of space in the structure of efficient VWM representations, in the current study we ask whether space is fundamental to SI VWM representations as well. Results from a recent SI VWM study identified a robust spatial structure when space was task relevant and the entire spatial context was constant throughout the experiment (Magen and Berger-Mandelbaum, 2018). The current study takes a further step in understanding the role of space in SI VWM, by testing whether a spatial structure would still be present in SI VWM when space was task irrelevant.

Magen and Berger-Mandelbaum (2018) explored the structure of SI VWM representations, using a modified change detection task. In each trial, participants were presented with a horizontal display of eight visual targets (either real-world objects or abstract shapes) from which they selected three or four targets they memorized and then placed them in several locations in a circular array of eight locations. On half of the trials one of the targets repeated. Following a short delay, participants were probed on object-location conjunctions, deeming space task relevant.

Verbal reports provided by the participants and analysis of the spatial configurations they constructed were used to uncover the strategies that guided them in the construction of the SI VWM representations. The results showed that abstract shapes were selected most frequently based on their resemblance to familiar objects that could be verbalized, while real world objects were mostly selected based on visual features such as color. While participants reported selecting visual targets based on these non-spatial features, their selections were spatially biased to targets presented on the left and central parts of the horizontal target display from which they selected the visual targets they memorized. Importantly, when faced with the circular array, participants placed the to-be memorized visual targets in structured spatial configurations, organized most frequently by the Gestalt organization cue of symmetry and to a lesser extent by cues of proximity and similarity. Participants also formed complex representations, which were based on the interaction of two Gestalt organization cues of proximity and similarity.

Notably, the construction of the SI VWM memory displays was time consuming. Reaction time (RT) for the first visual target or the first location of the sequences participants selected were longer relative to subsequent items in the sequence and increased with set size. These RT findings suggested that participants invested time in planning the memory displays they constructed before they executed their selections. Overall, the results of Magen and Berger-Mandelbaum (2018) suggested that participants have access to the metacognitive knowledge on the benefit of structure (based on Gestalt cues) in VWM and invested time and resources during encoding to construct spatially structured displays in order to maximize maintenance and retrieval processes.

In the current study, we explored how fundamental is space in the structure of SI VWM representations. Unlike our previous study (Magen and Berger-Mandelbaum, 2018), space was irrelevant during retrieval, and the spatial context varied randomly between trials. We assumed that if participants have a metacognitive knowledge on the spatial structure of representations in VWM, they would invest resources in constructing spatially structured memory representations, although the task emphasized only non-spatial visual information. Note that in this respect, SI VWM deviates considerably from non-SI (provided) VWM. While the spatial configuration is an emergent property that is easily extracted from the visual display when the memory displays are provided to the participants (Jiang et al., 2000), building such representations is time consuming (Magen and Berger-Mandelbaum, 2018; Magen and Emmanouil, 2018). Moreover, the visual targets in the current study were distributed randomly across the display, and therefore imposing a spatial structure on the selected targets could potentially constrain the use of non-spatial strategies.

Our main analysis in this study focused on the spatial and non-spatial characteristics of the memory representations that participants constructed (see section “Experiment 1” for details). Construction of spatially structured memory representations would suggest that participants have access to the metacognitive knowledge on the fundamental role of space in VWM. Nevertheless, the construction of such representations would not reveal whether that knowledge is implicit or explicit. A strategy questionnaire that participants filled out and manipulations introduced in Experiment 2 explored the extent to which this metacognitive knowledge was explicit and could be strategically manipulated.

## Experiment 1

The goal of Experiment 1 was to examine whether participants would construct spatially structured memory representations in a SI VWM task, in which space was task irrelevant. Participants were presented on each trial with displays of 12 randomly distributed pictures of real world objects. In the SI encoding condition, they were asked to select 1–7 pictures to memorize. An additional non-SI (i.e., provided) condition was introduced in the task, in which participants memorized 1–7 pictures that were randomly selected for them by the computer. Following a short delay, participants were probed on a single central target and indicated whether it matched or not one of the memorized items. The spatial structure formed between the targets selected in the SI condition was evaluated and was compared to the spatially unstructured non-SI representations.

As in our previous studies (Magen and Emmanouil, 2018; Milchgrub and Magen, 2018), spatial structure was defined based on a body of literature on provided (non-SI) spatial WM, which had identified the main characteristics of structured spatial configurations that benefited memory performance. Because these characteristics are relevant for the current study, we describe them here in detail. Note that thus far we have used the term spatial configuration to describe the spatial structure inherent in memory displays. In the context of the present study (and previous studies on spatial WM), we will also use the term spatial sequence to capture the dynamic construction process of the spatial configurations.

The literature has shown that structured spatial sequences that were based on familiar shapes, or followed well-established perceptual Gestalt organization cues of proximity, good continuation, symmetry, and linearity benefited memory performance (Kemps, 2001; Bor et al., 2003; De Lillo, 2004; Parmentier et al., 2005). Specifically, two characteristics of the spatial sequence path, an imaginary line between two successive to-be-remembered locations in the sequence, were shown to have an impact on memory performance. One of these characteristics is the path length, defined as the distance between two successive locations in the sequence. Sequences with longer paths were correlated with poorer memory performance, a finding known as the path length effect (Parmentier et al., 2005; Guerard et al., 2009; Guerard and Tremblay, 2012). In addition, the path complexity, reflected in the number of path crossings (i.e., the number of times that a path between two successive locations crosses another path between two other locations), has been found to have an impact on performance as well. Memory accuracy was reduced as the number of path crossings increased (Kemps, 2001; Parmentier et al., 2005). Note that path characteristics have a temporal dimension as well as a spatial one. Nevertheless, the temporal order is determined by spatial considerations of proximity and complexity, and path characteristics have a direct impact on spatial WM performance, even when participants are probed on one location. Studies that found enhanced memory performance for structured spatial sequences explained the performance benefits in terms of grouping. Locations in structured spatial sequences were easily grouped into higher-order spatial configurations, whereas the locations in spatially unstructured sequences often disrupted grouping and consequently memory performance (Parmentier et al., 2005; Guerard et al., 2009).

We recently used these characteristics to evaluate the spatial configurations participants constructed in a spatial SI WM task (Magen and Emmanouil, 2018; see also Milchgrub and Magen, 2018). Participants in this task selected the spatial locations they memorized from an array of locations that were distributed randomly across the display. Performance in the spatial SI WM task was compared to a non-SI task, in which participants memorized random spatial sequences that were provided to them. The results revealed that relative to random sequences, the constructed SI spatial sequences had a shorter average path length, consisted of fewer path crossings, and followed more frequently simple and linear shapes. The structured SI representations demonstrated that participants had access to metacognitive knowledge on the benefits of structure in spatial WM. Analysis of encoding RT showed that constructing these sequences involved planning and demanded resources. RT for the first location in the selected spatial sequence increased relative to subsequent locations and increased further with the sequence length. This pattern was absent from the non-SI condition, in which participants encoded provided spatial sequences. Finally, accuracy in the SI condition was higher than in the non-SI condition, even when the structure of the SI and non-SI spatial sequences was matched, demonstrating that self-initiation benefited performance beyond the benefit of structure.

The characteristics of spatially structured SI memory representations identified by Magen and Emmanouil (2018) were used to evaluate spatial organization in the current study as well. In addition, at the end of the experiment, we asked participants to describe the strategies they used to select the memory displays (cf. Magen and Berger-Mandelbaum, 2018). We predicted that if participants intended to memorize the spatial configuration of the memory displays they constructed, they would form spatially structured sequences. Otherwise, selection of targets based solely on non-spatial strategies should result in unstructured random spatial configurations.

### Methods

#### Participants

Twenty-four students from the Hebrew University participated in a 1-h session. They provided informed consent before participating in the study for course credit or payment. The study was approved by the Hebrew University IRB.

#### Stimuli and Design

Participants sat in a dimly lit room at a distance of 100 cm from the display and rested their head on a chin rest. In the SI and non-SI encoding conditions, participants were presented with an array of 12 pictures of real world objects (each picture measuring 1.16° × 1.16° of visual angle, presented on a gray background) appearing jittered (up to 0.19° of visual angle) in random locations within an invisible 6 × 6 matrix (measuring 10.32° × 10.32° of visual angle). A pool of 504 pictures of real world objects from different categories (e.g., fruits, furniture, toys, and household objects) was used in the study for the SI and non-SI encoding conditions. Pictures were selected randomly on each trial, with the limitation that each picture could appear only once in each experimental block. A large set of targets was used such that different combinations of pictures would appear on each trial for each participant and would not direct participants to prefer certain strategies over others. For the same reason, the pictures were not controlled for low level features. Of the 12 presented pictures, participants memorized 1–7 pictures, selected by them in the SI condition, or selected randomly by the computer and provided to them in the non-SI condition. The set size manipulation was blocked, and block order was randomized across participants.

A single probe appeared at the center of the screen in both the SI and non-SI conditions. In the match condition (50% of the trials), the probe matched one of the selected targets, with equal frequency for targets at each serial position of the sequences selected during encoding. For instance, when three targets were selected and memorized, one third of the matched probes matched the target selected first, one third matched the target selected second, and the remaining trials matched the target selected third. In the non-match trials (remaining 50% of the trials), the probe was one of the targets in the original array that were not selected on that trial.

#### Procedure

**Figure 1:**
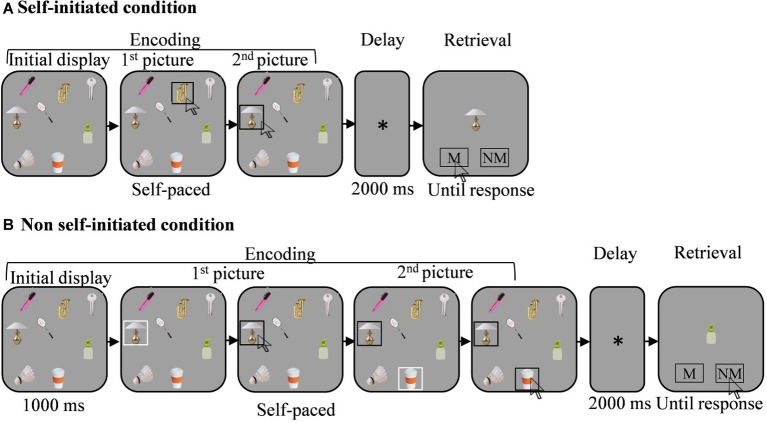
Illustration of the memory task (see text for further detail). Each trial began with the presentation of an initial spatial array of 12 pictures (for presentation purposes only eight pictures appear in the figure. Note that the pictures are not to scale). In the self-initiated (SI) condition **(A)** participants clicked sequentially on the pictures they wished to memorize. In the non-SI condition **(B)** participants clicked in sequence on the pictures selected randomly by the computer, which were marked by a white frame. Encoding was self-paced in both encoding conditions. In both conditions, encoding was followed by a short delay of 2,000 ms, after which a single probe appeared in the screen center, and either matched or did not match one of the selected pictures. Participants responded by clicking on one of the response areas that appeared with the probe. M, match. NM, non-match.

In both the SI and non-SI conditions, the trial started with the onset of a fixation cross for 500 ms, followed by the appearance of the target display (see Figure 1). In the SI condition, participants selected 1–7 targets sequentially, by clicking each selected target with the left key of the computer mouse. Following each selection, a black frame appeared around the selected target marking its selection. Encoding was self-paced.

In the non-SI condition, participants memorized 1–7 targets that were provided to them. In each trial, a white frame appeared around the 1–7 targets, one at a time, marking the target the computer selected. To equate the motoric response to the SI condition, the trial proceeded only after participants clicked each marked target in sequence. In response, the white frame turned black marking the selected target. Consequently, similar to the SI condition, participants controlled the pace and the duration of the encoding phase in the non-SI condition as well. In the non-SI condition, the first target in the sequence appeared 1,000 ms after the onset of the initial display. This was done to allow participants to scan the targets in the display before the first item was selected by the computer, as we hypothesized they would do in the SI encoding condition. In addition, a 200 ms delay was introduced in the non-SI condition between the selection (i.e., click) of the current target and the appearance of the white frame which marked the next to-be memorized target in the sequence.

In both the SI and the non-SI conditions, all chosen locations remained visible until the end of the encoding phase and disappeared 200 ms after the last location was clicked. The selected items remained visible throughout the trial to equate the conditions in the SI and non-SI conditions. We assumed that participants would plan their selections in the SI condition and therefore would hold the entire display in mind during encoding. Keeping the entire display visible during encoding in the SI and non-SI conditions reduced this potential advantage of self-initiation.

The maintenance and retrieval phases were identical in the SI and the non-SI conditions. Encoding was followed by a delay phase of 2,000 ms during which a fixation cross appeared at the screen center and which in turn was followed by the appearance of the central probe. The probe either matched or did not match one of the selected targets. Responses were registered by clicking with the mouse on one of two gray rectangles (measuring 0.69° × 1.72° of visual angle in height and width) with the words match and non-match written on them in Hebrew. The rectangles were presented at the bottom of the screen (3.72° of visual angle below fixation) along with the probe. Accuracy was stressed in both the SI and non-SI conditions.

In each encoding condition, there were seven blocks of approximately 12 trials, one in each set size. There were 12 trials in set sizes 1, 2, 3, and 6, 16 trials in set size 4, 10 trials in set size 5, and 14 trials in set size 7. The number of trials varied between set sizes, because the probes in the match condition (half of the trials in each set size) appeared equally often in each of the serial order positions in the memory array.

Each participant performed the SI and the non-SI tasks in two separate blocks within the same session, with task order counterbalanced across participants. Each block (SI or non-SI) began with four short practice blocks consisting of two trials each, for set sizes 1–4. Throughout the experiment, an error message was presented on the screen for 500 ms following an incorrect response. The intertrial interval (ITI) was 1,500 ms in correct and error trials.

#### Strategy Questionnaire

At the end of the experiment, participants filled out a questionnaire with two open-ended questions. The first question asked them to detail the strategies they used to select the targets in the SI condition, while the second question asked whether and how these strategies benefited memory performance.

### Results

Our analysis focused on three aspects of the results, encoding RT, the strategies participants reported using in selecting the targets they memorized and the characteristics of the spatial configurations they constructed. An additional analysis focused on accuracy.

#### Encoding Reaction Time

**Figure 2:**
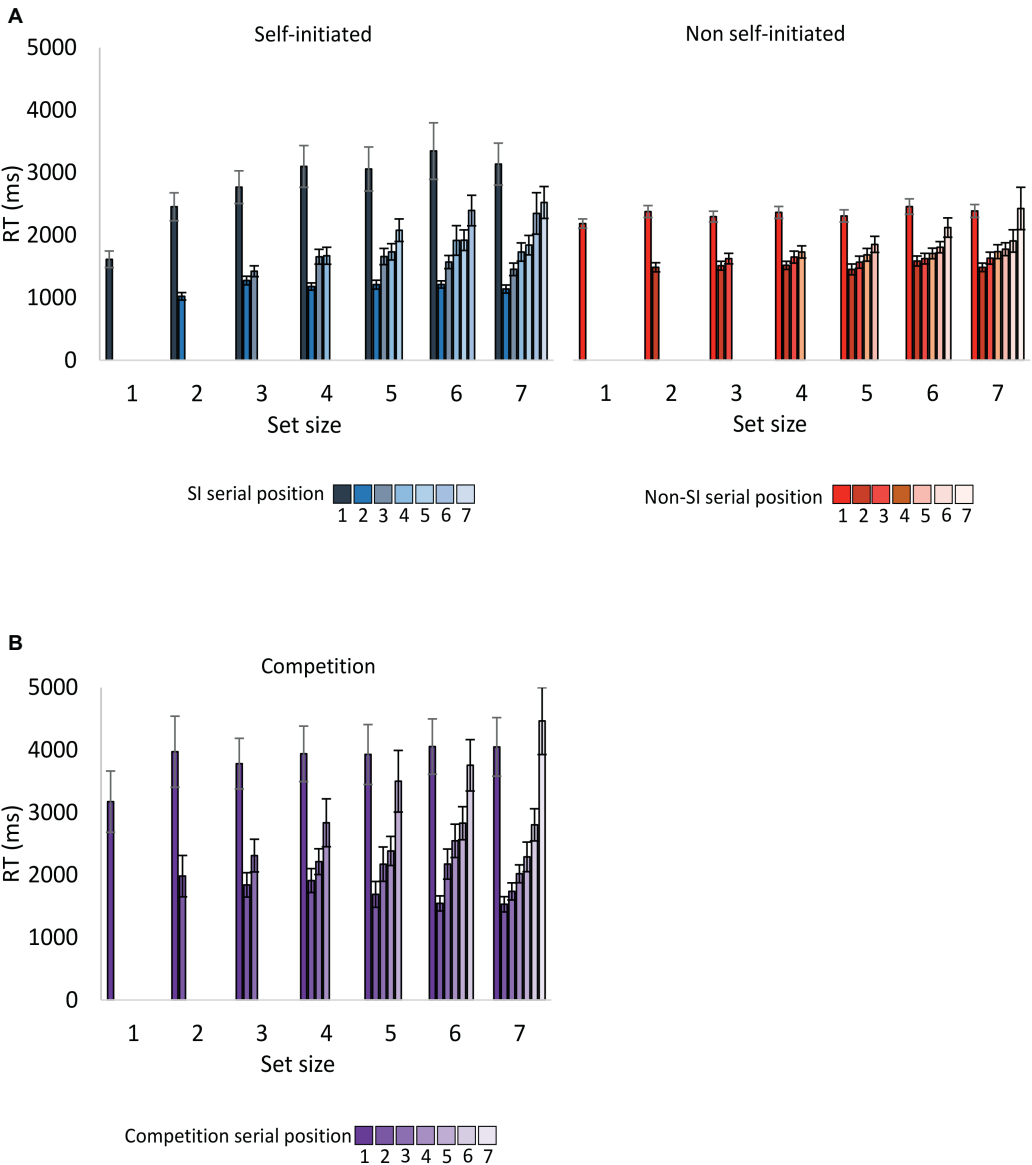
Encoding RT as a function of encoding condition, serial position and set size for **(A)** Experiment 1 **(B)** Experiment 2. Except for the first target, encoding RT for each target was calculated with respect to the selection of the previous target in the sequence. Error bars represent standard error of the mean. SI, self-initiated.

RTs for the entire sequence are presented in Figure 2. Given our previous studies (Magen and Emmanouil, 2018; Milchgrub and Magen, 2018), the analysis of encoding RT focused on RT for the first target in the sequence, which reflected planning. We also analyzed RT for the last target in the sequence, which showed how long participants took to review the target display before it disappeared, which reflected the participants perceived complexity of the array. In our previous studies, the non-SI condition yielded longer RTs for the last target in the sequence.

##### Reaction Time for the First Target in the Sequence

A repeated-measures ANOVA was conducted with the within-participant factors of encoding condition (SI and non-SI) and set size (1–7). The main effect of encoding was not significant *F*(1,23) = 3.59, *p* = 0.07, $\eta_{p}^{2}$ = 0.135, whereas the main effect of set size was significant *F*(6,138) = 10.58, *p* < 0.001, $\eta_{p}^{2}$ = 0.315. The interaction between set size and encoding was significant as well *F*(6,138) = 6.49, *p* < 0.001, $\eta_{p}^{2}$ = 0.220. Follow-up ANOVAs for each encoding condition revealed a significant set size effect in the SI condition *F*(6,138) = 9.45, *p* < 0.001, $\eta_{p}^{2}$ = 0.291 (explained by linear and quadratic contrasts, *F*s(1,23) = 19.51, 11.45, *p*s < 0.01, $\eta_{p}^{2}$ = 0.459, 0.332, respectively), and a non-significant effect in the non-SI condition *F*(6,138) = 1.53, *p* = 0.17, $\eta_{p}^{2}$ = 0.062. Thus, in the SI condition, RT for the first target increased with set size (up to set size 4), which suggests that participants planned the sequence of targets they selected before its execution and therefore required more time as set size increased.

##### Reaction Time for the Last Target in the Sequence (Set Sizes 2–7)

RT for the last target in the sequence was calculated with respect to the selection of the previous target in the sequence. The results yielded a non-significant main effect of encoding condition *F*(1,23) = 0.09, *p* = 0.77, $\eta_{p}^{2}$ = 0.004. The main effect of set size was significant *F*(5,115) = 15.21, *p* < 0.001, $\eta_{p}^{2}$ = 0.398, and interacted significantly with encoding *F*(5,115) = 2.99, *p* < 0.05, $\eta_{p}^{2}$ = 0.115. Follow-up ANOVAs for each encoding condition revealed significant set size effects in the SI and non-SI conditions, *F*s(5,115) = 19.45, 5.19, *p*s < 0.001, $\eta_{p}^{2}$ = 0.458, 0.184, respectively. The interaction between encoding and set size reflected the findings that in the lower set sizes, RT for the last target in the sequence was longer in the non-SI condition, whereas in the higher set sizes, RT was longer in the SI condition.

#### Encoding Strategies

The analysis of encoding strategies focused on the participants’ responses to the strategy questionnaire and on the main characteristics of the spatial configurations they constructed.

##### Strategy Questionnaire

The type of strategies and the number of participants who selected each of these strategies are presented in Table 1.

**Table 1:** The number of participants who selected the different encoding strategies in Experiments 1 and 2.

Strategies	Visual	Semantic	Story	Self-reference	Spatial
**Experiment 1**					
SI	14	17	5	7	3
**Experiment 2**					
Competition	14^*^	8^*^	1^*^	3	0
Competition-SI	6	5	5	1	0

*The results of Experiment 2 are presented separately for participants who selected strategies to disrupt memory performance (competition, *n* = 16) and those who reported selecting targets to enhance memory performance (competition-SI, *n* = 8) (see text for further details)*.

*SI, self-initiated*.

* *Used these strategies in the opposite way, to disrupt grouping (see the “Results” section of Experiment 2, for further details)*.

Participants used on average 1.92 strategies (SD = 0.78, Mode = 2, Range 1–4). Overall, five different strategies guided participants in their selections. Most frequently, participants selected targets based on semantic categories or on visual features (e.g., targets with similar colors or shapes or targets with distinct visual features). Five participants reported that they selected targets that fitted a story they created and three participants selected targets with reference to themselves. Of the 24 participants, only three participants reported using spatial strategies, by selecting targets positioned in close spatial proximity.

In their responses to the second question in the questionnaire, namely whether the strategy they used facilitated memory performance, all participants responded positively. They explained that the strategies they employed reduced the amount of information they memorized and helped identify matched and non-matched probes (by excluding targets that did not fit the semantic or visual regularities they set, or the story they created). Several participants explained specifically that it was easier to memorize visually distinct targets.

##### Spatial Structure Analysis

**Figure 3:**
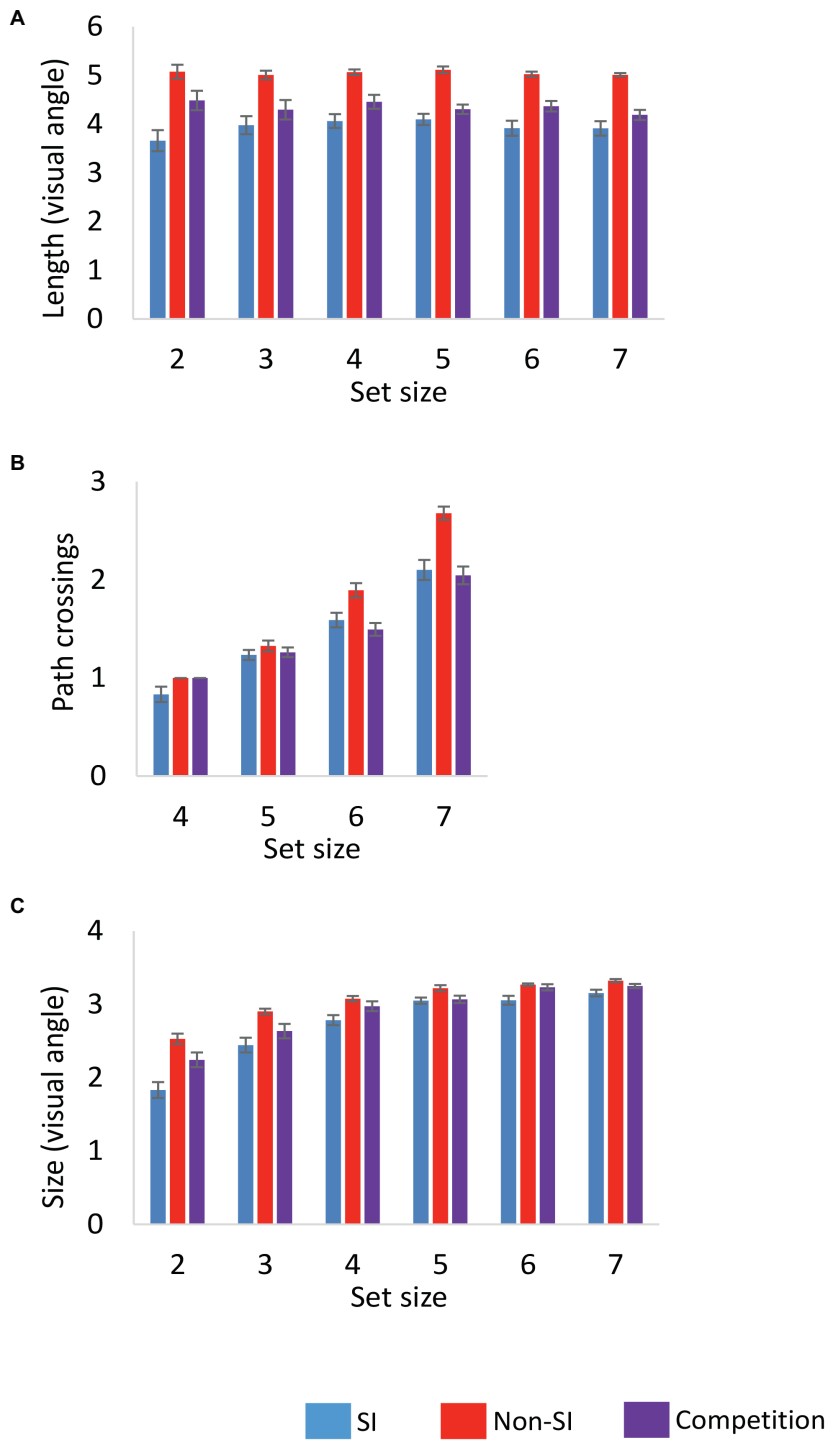
Characteristics of the spatial configurations as a function of encoding condition and set size in Experiments 1 and 2: **(A)** the mean path length, **(B)** the number of path crossings, and **(C)** configuration size. Error bars represent standard error of the mean. SI, self-initiated.

Our next analysis focused on the characteristics of the spatial configurations that participants constructed, comparing them to the random computer-generated configurations. First, two characteristics of the path were analyzed, the average path length and the number of path crossings. An additional analysis examined the size of the spatial configuration. The results are presented in Figure 3.

We also explored the direction of movements participants made when they selected the sequence of targets during encoding, to reveal the overall shape of the spatial sequence, and whether participants tended to initiate the spatial sequence at the top and left side as shown in previous studies (Magen and Emmanouil, 2018; Milchgrub and Magen, 2018). The results are presented in Table 2.

**Table 2:** Percent of movement directions in the construction of the spatial sequences in Experiments 1 and 2, for each encoding condition and set size, separately for the horizontal and vertical axes.

Set size	Condition	Horizontal			Vertical		
		Left	Right	None	Down	Up	None
**Experiment 1**							
2	SI	37.85	42.71	19.44	33.68	40.63	25.69
	Non-SI	37.15	46.18	16.67	42.01	43.06	14.93
3	SI	38.89	40.80	20.31	42.01	35.76	22.22
	Non-SI	45.14	39.93	14.93	44.62	41.84	13.54
4	SI	41.06	40.45	18.49	39.84	37.24	22.92
	Non-SI	40.89	43.40	15.71	42.45	42.97	14.58
5	SI	39.90	42.08	18.02	41.56	35.73	22.71
	Non-SI	43.65	43.33	13.02	44.79	42.50	12.71
6	SI	38.82	42.50	18.68	40.28	35.83	23.89
	Non-SI	43.19	42.29	14.51	41.88	42.71	15.42
7	SI	39.09	40.48	20.44	41.17	35.86	22.97
	Non-SI	43.40	42.06	14.53	42.81	43.40	13.79
**Average SI**		39.27	41.50	19.23	39.76	36.84	23.40
**Average non-SI**		42.24	42.87	14.90	43.09	42.75	14.16
**Experiment 2**							
2	Competition	44.44	40.63	14.93	38.89	38.54	22.57
3	Competition	37.67	44.79	17.53	40.28	38.54	21.18
4	Competition	37.33	44.01	18.66	42.27	38.45	19.27
5	Competition	40.31	42.92	16.77	42.40	38.65	18.96
6	Competition	40.07	43.47	16.46	41.11	39.79	19.10
7	Competition	39.68	42.76	17.56	41.67	38.24	20.09
**Average competition**		39.92	43.10	16.99	41.10	38.70	20.20

*SI, self-initiated*.

###### Path Length (Set Sizes 2–7)

The average path length was significantly shorter in the SI condition as indicated by a main effect of encoding *F*(1,23) = 87.34, *p* < 0.001, $\eta_{p}^{2}$ = 0.792. The main effect of set size and its interaction with encoding were non-significant *F*(5,115) = 1.01, 1.48, *p*s = 0.41, 0.20, $\eta_{p}^{2}$ = 0.042, 0.060, respectively.

###### Path Crossings (Set Sizes 4–7)

The number of path crossings was significantly smaller in the SI condition relative to the non-SI condition *F*(1,23) = 34.64, *p* < 0.001, $\eta_{p}^{2}$ = 0.602. The main effect of set size was also significant *F*(3,69) = 146.86, *p* < 0.001, $\eta_{p}^{2}$ = 0.865, as was its interaction with encoding *F*(3,69) = 6.27, *p* < 0.001, $\eta_{p}^{2}$ = 0.214. Follow-up ANOVAs showed that the effect of set size was significant in both the SI and the non-SI conditions, *F*s(3,69) = 50.42, 158.68, *p*s < 0.001, $\eta_{p}^{2}$ = 0.687, 0.873, respectively. The interaction between encoding and set size resulted from a larger increase in the number of path crossings with set size in the non-SI condition.

###### Configuration Size (Set Sizes 2–7)

In addition to path characteristics, we included a measure of the overall size of the spatial configuration. Very different paths (in terms of length and the number of crossings) can be formed between locations in the very same spatial configuration, and therefore these characteristics by themselves do not indicate whether participants selected targets in close proximity, relative to the random displays. The overall size of the spatial configurations was determined by calculating the centroid between all the locations in the configuration and then measuring the distance between each of the locations to the centroid. The analysis was based on the average distance between the centroid and all the targets in the configuration.

The average overall size of the spatial configurations was significantly smaller in the SI condition as reflected in a main effect of encoding *F*(1,23) = 47.87, *p* < 0.001, $\eta_{p}^{2}$ = 0.675. The main effect of set size was also significant *F*(5,115) = 89.31, *p* < 0.001, $\eta_{p}^{2}$ = 0.795, as the size of the configurations increased with set size in both encoding conditions. The interaction of set size with encoding was also significant *F*(5,115) = 8.95, *p* < 0.001, $\eta_{p}^{2}$ = 0.280, as the difference between the two encoding conditions decreased as set size increased, probably due to a ceiling effect on the overall size of the configuration as the number of locations in the sequence increased. Follow-up ANOVAs showed that the set size effect was significant in the SI *F*(5,115) = 60.29, *p* < 0.001, $\eta_{p}^{2}$ = 0.724 and in the non-SI conditions *F*(5,115) = 49.36, *p* < 0.001, $\eta_{p}^{2}$ = 0.682.

###### Direction of Movements

The final analysis examined the overall shape of the self-initiated spatial sequences, by examining the direction of movements in the horizontal and vertical axes. The movements were also analyzed in the non-SI condition for comparison. Each movement was scored on both the horizontal and the vertical axes. On the horizontal axis, movements were divided into left, right, and no horizontal movements (i.e., straight vertical movements), and in the vertical axis, movements were divided into down, up, and no vertical movements (i.e., straight horizontal movements). The analyses were motivated by the results of our previous studies (Magen and Emmanouil, 2018; Milchgrub and Magen, 2018). First, the number of straight vertical and horizontal movements were compared between the SI and non-SI conditions. Second, to examine whether movements were initiated at the top and left side, we created a difference score for each axis and compared these scores across the two encoding conditions. As can be seen in Table 2, the pattern of movements in the SI spatial sequences deviated from the random movements generated in the non-SI condition. Movements in the same horizontal or vertical axis (creating straight lines) were more frequent in the SI condition. An ANOVA with encoding and set size revealed a main effect of encoding, *F*(1,23) = 66.59, *p* < 0.001, $\eta_{p}^{2}$ = 0.743 showing a larger percent of straight horizontal movements in the SI condition. The main effect of set size and its interaction with encoding were not significant *F*s < 1. The same pattern was observed for the straight vertical movements, showing a significant main effect of encoding *F*(1,5) = 21.56, *p* < 0.001, $\eta_{p}^{2}$ = 0.484, whereas the main effect of set size and its interaction with encoding were not significant *F*s < 1. Thus, the SI configurations consisted of more linear shapes.

Two additional ANOVAs explored whether vertical (more downward than upward movements) or horizontal (more rightward than leftward movements) biases were present in the SI configurations, by comparing the difference scores between the SI and non-SI conditions. The two ANOVAs yielded null effects. The vertical bias yielded non-significant effects of encoding, set size, and a non-significant interaction between them, all *F*s < 1.54, *p*s > 0.18, $\eta_{p}^{2}$ < 0.063. The ANOVA examining the horizontal bias revealed a non-significant main effect of encoding *F* < 1. The main effect of set size was significant, mainly due to random variations in movements in the non-SI condition (see Table 2), *F*(1,5) = 2.49, *p* < 0.05, $\eta_{p}^{2}$ = 0.098. The interaction between encoding and set size was non-significant *F* < 1.

#### Accuracy

**Figure 4:**
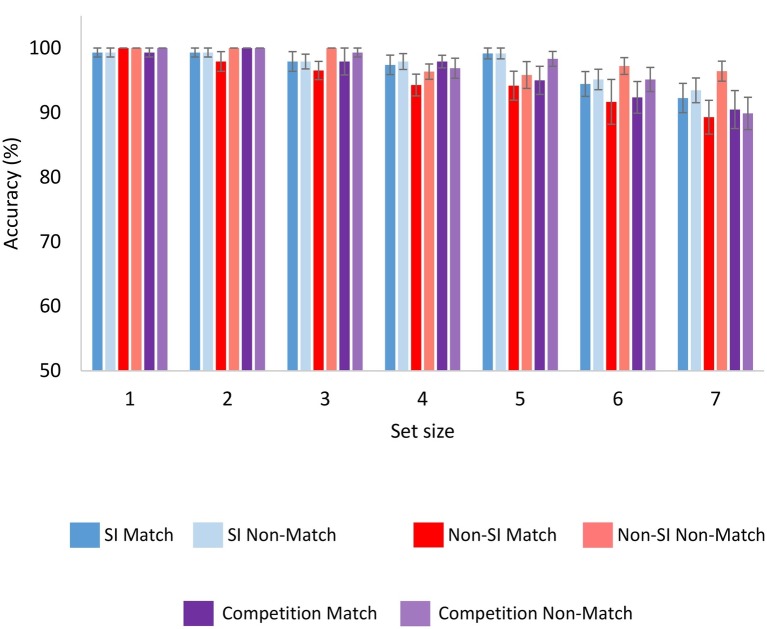
Accuracy as a function of encoding condition, set size and probe condition (match or non-match) for Experiments 1 and 2. Error bars represent standard error of the mean. SI, self-initiated.

Accuracy in the match and non-match trials was compared across the SI and non-SI encoding conditions. Accuracy in the match condition was averaged across all serial positions in each set size, to obtain a single measure of accuracy. As evident from Figure 4, although accuracy was almost at ceiling, there were small differences between the different conditions. A repeated-measured ANOVA with encoding, set size, and probe condition (match or non-match) as within-participant factors showed non-significant main effects of encoding and probe *F*(1,23) = 2.31, *p* = 0.14, $\eta_{p}^{2}$ = 0.091 and *F*(1,23) = 3.42, *p* = 0.08, $\eta_{p}^{2}$ = 0.129, respectively. Nevertheless, the interaction between them was significant, *F*(1,23) = 5.76, *p* < 0.05, $\eta_{p}^{2}$ = 0.200. The main effect of set size was significant *F*(6,138) = 10.57, *p* < 0.001, $\eta_{p}^{2}$ = 0.315 and did not interact significantly with encoding *F*(6,138) = 1.58, *p* = 0.16, $\eta_{p}^{2}$ = 0.064, or probe *F*(6,138) = 1.08, *p* = 0.38, $\eta_{p}^{2}$ = 0.045. The three-way interaction of encoding, probe, and set size was non-significant *F*(6,138) < 1.

To follow-up on the interaction between the encoding and probe factors, two additional ANOVAs were conducted separately for the match and non-match trials. The analysis showed a significant main effect of encoding in the match trials *F*(1,23) = 4.86, *p* < 0.05, $\eta_{p}^{2}$ = 0.174, but not in the non-match trials *F* < 1. Thus, accuracy was significantly higher in the SI condition, but only in match trials when a target from the memory display appeared as the probe.

### Discussion

The results of Experiment 1 demonstrated that the spatial sequences participants constructed were spatially structured relative to the non-SI sequences, although space was task irrelevant. Relative to the non-SI condition, the memory displays constructed in the SI condition were smaller in size and were characterized by a shorter average path length, by a smaller number of path crossings and by more linear shapes. We assume that participants imposed the spatial structrue on the memory representations they constructed, to benefit memory performance, rather than, for instance, ease the selection process during encoding. We assume that structure was intended to benefit memory for several reasons. First, encoding was self-paced, and participants could spend as much time as they needed to familiarize themselves with each target. It seems unlikely therefore, that they would impose a spatial structure during encoding only for selection purposes, structure that could disrupt the implementation of the non-spatial encoding strategies. Most importantly, the spatial structure that participants imposed in this study is consistent with the structure that has been shown in previous non-SI and SI studies to benefit memory performance (e.g., Kemps, 2001; Parmentier et al., 2005; Magen and Emmanouil, 2018). Consistent with our previous studies (Magen and Berger-Mandelbaum, 2018; Magen and Emmanouil, 2018; Milchgrub and Magen, 2018), the constructed configurations were the result of effortful planning as reflected by encoding RT for the first target in the sequence, which increased with set size only in the SI condition.

Accuracy was high in both encoding conditions demonstrating that participants adjusted encoding RT to maximize memory performance in both conditions. Nevertheless, accuracy was still significantly higher in the SI condition when the probe matched one of the memorized items. The high and similar accuracy levels could explain why, unlike our previous studies, RT for the last target in the sequence was similar in the two encoding conditions. Perhaps the nature of the stimuli in the task, in addition to the unlimited encoding time, allowed participants to encode items in the SI and the non-SI conditions such that the perceived difficulty of the maintained memory representations was similar across the two encoding conditions.

Participants’ reports on the strategies they used revealed that the most frequently reported strategies were non-spatial, grouping items based on semantic categories or visual features. Spatial strategies were scarcely reported, which suggests that participants were largely unaware of the spatial characteristics of the memory representations they formed. Remember that the visual targets were distributed randomly across the display on each trial, and therefore it is unlikely that the visual targets that fitted the participants’ non-spatial strategies were consistently placed in close proximity. Thus, the construction of the spatially organized configurations must have constrained the implementation of the non-spatial strategies to some degree.

The non-spatial visual strategies used to group the memory targets were similar to the strategies identified in our previous study (Magen and Berger-Mandelbaum, 2018), although in that study participants based their selections of real-world objects more frequently on visual features than on semantic categories. The limited set size conditions of 3 or 4 items in our previous study could explain this difference. It is possible that participants have found it necessary to employ diverse strategies in this study, as set size increased to seven items. This suggestion is supported by the finding that the strategies of self-reference and the creation of a story were only used in the current study and not in the study of Magen and Berger-Mandelbaum (2018).

Taken together, the results of Experiment 1 suggest that, similar to our previous studies in which space was task relevant, participants have metacognitive knowledge on the role of space in VWM. This knowledge was nevertheless mostly implicit, as only a handful of participants reported spatial proximity as one of the strategies they used to construct their memory representations. Note that in our previous study (Magen and Berger-Mandelbaum, 2018), when space was relevant to task performance, participants provided verbal descriptions of the spatial strategies they used to place the targets on the circular array they were presented with during encoding. Thus, spatial strategies by themselves could be explicit in other tasks.

Experiment 2 utilized a non-verbal manipulation to uncover whether these spatial strategies could be manipulated flexibly, although the metacognitive knowledge that guided them was implicit.

## Experiment 2

The main purpose of Experiment 2 was to explore whether participants would construct less structured spatial configurations under opposite instructions. Participants in this task were asked to construct memory displays for a hypothetical competitor in a memory contest, displays that in effect would disrupt memory performance. We had used this manipulation in the past to reveal the flexibility of spatial SI memory representations (Magen and Emmanouil, 2018). The results of this experiment showed that when asked to disrupt memory performance, participants constructed spatial sequences that were characterized by a longer average path length, a larger number of path crossings, and by more non-linear shapes, relative to a SI encoding condition. Thus, in the “competition” task, participants manipulated the same characteristics that they used to construct structured SI representations, revealing flexible use of the metacognitive knowledge on the impact of these characteristics on memory performance.

Experiment 2 used the “competition” manipulation with the SI VWM task of Experiment 1, to examine whether participants would manipulate the spatial characteristics of the visual memory representations they would construct for a hypothetical competitor. Similar to Experiment 1, we also asked participants to provide verbal reports on the strategies that guided the selection of the targets they memorized.

### Methods

#### Participants

A new group of 24 students from the Hebrew University participated in a 30-min session for course credit or payment. Participants provided informed consent before participating in the study. The study was approved by the Hebrew University IRB.

The task was identical to the SI condition in Experiment 1, except for task instructions, which asked participants to select locations for a hypothetical competitor in a memory contest. As in Experiment 1, participants filled out a strategy questionnaire at the end of the experiment, in which they were asked to detail the strategies they used to select the targets during encoding. In Experiment 2, the participants were asked in addition whether and how the strategies they selected disrupted memory performance.

### Results

The main analysis focused on comparing the results of the competition condition in Experiment 2, to each of the SI and non-SI conditions in Experiment 1, in terms of encoding RT, the spatial and non-spatial encoding strategies they used, and accuracy.

As detailed in subsequent sections, the analysis of participants responses to the strategy questionnaire showed that 8 of the 24 participants reported using strategies with the attempt to enhance memory performance rather than disrupt it. These participants were not removed *post hoc* from the main analysis; however, at the end of the “Results” section, we included an additional analysis that compared the results of these 8 participants to the remaining 16 participants who followed the competition instructions.

#### Comparison of Experiments 1 and 2

##### Encoding Reaction Time

###### Reaction Time for the First Target in the Sequence

As evident in Figure 2 and confirmed in the analyses below, overall RT for the first target in the sequence was longer in the competition condition relative to the SI and the non-SI conditions. The first of two mixed-effects ANOVAs with set size as a within-participant factor and encoding condition (competition and SI) as a between-participant condition revealed a significant main effect of encoding *F*(1,46) = 5.41, *p* < 0.05, $\eta_{p}^{2}$ = 0.105, a significant main effect of set size *F*(6,276) = 5.81, *p* < 0.001, $\eta_{p}^{2}$ = 0.112, and a non-significant interaction between them, *F* < 1. The second ANOVA that compared the competition and the non-SI conditions showed a main effect of encoding *F*(1,46) = 15.22, *p* < 0.001, $\eta_{p}^{2}$ = 0.249, a non-significant main effect of set size *F*(6,276) = 1.50, *p* = 0.18, $\eta_{p}^{2}$ = 0.031, and a non-significant interaction between them *F* < 1.

The results of Experiment 1 showed that RT for the first target in the sequence increased with set size in the SI condition but not in the non-SI condition. The results of the competition condition were similar to the non-SI condition, revealing a non-significant main effect of set size *F*(6,138) < 1. Nevertheless, when the competition and the SI conditions were compared, the two-way interaction of set size and encoding was non-significant. This is most likely due to the inclusion in the data of Experiment 2, the group of participants who adopted an SI encoding strategy rather than a “competition” strategy (see below).

###### Reaction Time for the Last Target in the Sequence (Set Sizes 2–7)

Comparing the competition and the SI conditions yielded a main effect of encoding *F*(1,46) = 14.22, *p* < 0.001, $\eta_{p}^{2}$ = 0.236, as RT for the last target in the sequence was longer in the competition condition relative to the SI condition. The main effect of set size was also significant *F*(5,230) = 22.45, *p* < 0.001, $\eta_{p}^{2}$ = 0.328 and did not interact significantly with encoding *F*(5,230) = 1.44, *p* = 0.24, $\eta_{p}^{2}$ = 0.030. RT for the last item was longer in the competition condition relative to the non-SI condition as well, as shown by a main effect of encoding *F*(1,46) = 14.38, *p* < 0.001, $\eta_{p}^{2}$ = 0.238. The main effect of set size was also significant *F*(5,230) = 15.13, *p* < 0.001, $\eta_{p}^{2}$ = 0.248 and interacted significantly with encoding *F*(5,230) = 3.39, *p* < 0.05, $\eta_{p}^{2}$ = 0.069, as the difference between the two conditions increased with set size.

#### Encoding Strategies

##### Strategy Questionnaire

The results of the strategy questionnaire are presented in Table 1. The results of the 8 participants who reported using strategies to enhance memory performance are presented separately from the results of the remaining 16 participants.

Overall, participants in Experiment 2 used on average 1.79 strategies (SD = 0.59, Mode = 2, Range 1–3). As shown in Table 1, participants who reported using encoding strategies to disrupt memory performance used similar strategies to the participants in the SI condition in Experiment 1, but in the opposite way. Specifically, they selected targets that were visually dissimilar, and non-distinct, and selected targets from different semantic categories. Participants who created a story reported that they associated targets to the story in a personal way that would be difficult for others to decipher. Finally, several participants selected targets related to themselves, assuming that it would not benefit the memory of others. None of the participants mentioned space as a strategy for target selection. Participants explained that the targets they selected disrupted memory performance because they were not easily associated with each other and therefore increased the load on memory.

The eight participants who selected targets to enhance memory performance used the same strategies and explanations as in the SI condition in Experiment 1 (see Table 1).

##### Spatial Structure Analysis

Overall, participants in Experiment 2 constructed memory representations that were less spatially structured compared to the SI condition in Experiment 1. The results are presented in Figure 3.

###### Path Length (Set Sizes 2–7)

Comparing the competition and the SI condition showed a significant main effect of encoding *F*(1,46) = 6.89, *p* < 0.05, $\eta_{p}^{2}$ = 0.130, as the average path length was longer in the competition condition. The main effect of set size and its interaction with encoding were not significant, *F* < 1, and *F*(5,230) = 1.69, *p* = 0.14, $\eta_{p}^{2}$ = 0.035, respectively. Comparing the competition to the non-SI condition also showed a significant main effect of encoding *F*(1,46) = 40.76, *p* < 0.001, $\eta_{p}^{2}$ = 0.470. Although the path length in the competition condition was longer than in the SI condition, it was still significantly shorter than in the non-SI condition. The main effect of set size and its interaction with encoding were not significant *F*s < 1.

###### Path Crossings (Set Sizes 4–7)

In contrast to the path length, the average number of path crossings was similar in the competition and SI conditions. The main effect of encoding was non-significant *F* < 1, while the main effect of set size was significant *F*(3,138) = 100.36, *p* < 0.001, $\eta_{p}^{2}$ = 0.686. The two factors did not interact significantly *F*(3,138) = 1.40, *p* = 0.25, $\eta_{p}^{2}$ = 0.030. The average number of path crossings was significantly smaller in the competition condition relative to the non-SI condition *F*(1,46) = 47.79, *p* < 0.001, $\eta_{p}^{2}$ = 0.510. The main effect of set size was significant as well *F*(3,138) = 191.76, *p* < 0.001, $\eta_{p}^{2}$ = 0.807, as was its interaction with encoding *F*(3,138) = 12.12, *p* < 0.001, $\eta_{p}^{2}$ = 0.209. The interaction reflected the observation that the difference between the competition and non-SI conditions increased with set size.

###### Configuration Size (Set Sizes 2–7)

The comparison of the configuration size between the competition and the SI conditions yielded a significant main effect of encoding *F*(1,46) = 7.91, *p* < 0.01, $\eta_{p}^{2}$ = 0.147, showing that participants constructed larger spatial configurations in the competition condition. The main effect of set size was significant as well *F*(5,230) = 104.28, *p* < 0.001, $\eta_{p}^{2}$ = 0.694, and its interaction with encoding was marginally significant *F*(5,230) = 2.27, *p* = 0.05, $\eta_{p}^{2}$ = 0.047. The difference between the two conditions decreased with set size, most likely due to a ceiling effect on the overall size of the spatial configurations. The competition condition differed significantly from the non-SI condition as well, as the spatial configurations in the competition condition were on average smaller than in the non-SI condition *F*(1,46) = 10.35, *p* < 0.01, $\eta_{p}^{2}$ = 0.184. The main effect of set size was significant *F*(5,230) = 90.49, *p* < 0.001, $\eta_{p}^{2}$ = 0.663, and it did not interact significantly with encoding *F*(5,230) = 2.03, *p* = 0.08, $\eta_{p}^{2}$ = 0.042.

###### Path Shape

We focus in this analysis only the percent straight vertical and horizontal movements, which showed differences between the SI and non-SI conditions in Experiment 1. As shown in Table 2, the percent of linear shapes in the competition condition was intermediate between the SI and non-SI conditions. Straight horizontal and vertical movements were more frequent in the SI condition than in the competition condition, as the main effect of encoding was significant in both ANOVAs, *F*(1,46) = 4.51, *p* < 0.05, $\eta_{p}^{2}$ = 0.089 and *F*(1,46) = 5.06, *p* < 0.05, $\eta_{p}^{2}$ = 0.099, respectively. The main effects of set size or their interaction with encoding were not significant in either ANOVA, all *F*s < 1.13. Comparison of the competition and non-SI conditions revealed more frequent straight horizontal and vertical movements in the non-SI condition, as reflected in the main effects of encoding in both ANOVAs, *F*(1,46) = 40.94, *p* < 0.001, $\eta_{p}^{2}$ = 0.471 and *F*(1,46) = 5.10, *p* < 0.05, $\eta_{p}^{2}$ = 0.100, respectively. The main effects of set size or their interaction with encoding were non-significant, all *F*s < 1.02.

#### Accuracy

Accuracy was compared between the competition condition and the SI and non-SI conditions, although accuracy in the competition condition may be more difficult to interpret, as participants memorized information that they selected with the intent to disrupt memory performance. Similar to Experiment 1, accuracy was high in the competition condition as well, (see Figure 4). The analyses showed that accuracy was the same in the competition and SI conditions, as the main effect of encoding was non-significant *F* < 1, and neither was the main effect of probe (match or non-match) *F*(1,46) = 1.12, *p* = 0.30, $\eta_{p}^{2}$ = 0.024. Only the main effect of set size was significant *F*(6,276) = 16.25, *p* < 0.001, $\eta_{p}^{2}$ = 0.261. None of the interactions between the three factors were significant, all *F*s < 1. The competition and the non-SI condition yielded similar accuracy levels as well, *F* < 1 for the main effect of encoding. The other two main effects of set size and probe were significant *F*(6,276) = 16.19, *p* < 0.001, $\eta_{p}^{2}$ = 0.260, and *F*(1,46) = 5.97, *p* < 0.05, $\eta_{p}^{2}$ = 0.115, respectively. None of the interactions were significant, all *F*s < 1.75, *p*s > 0.19.

#### Comparing Participants Based on Reported Strategies

In this section, we compared the data of the 8 participants who reported using encoding strategies to enhance memory performance to the remaining 16 participants who reported using strategies to disrupt performance. Because of the small number of participants in each group, we averaged the data across set sizes to obtain one measure of RT, structure, and accuracy from each participant. Except for accuracy, which was evaluated in a mixed-model ANOVA, the two groups of participants were compared by independent *t*-tests (all two-tailed). Figures depicting the full set of results appear in the Supplementary Figures SA1–SA3.

##### Reaction Time for the First Target in the Sequence

RTs for the first target in the sequence were longer among the participants who followed the competition instructions relative to the participants who selected memory displays to enhance memory performance (*M* = 4275.19, SD = 1868.41 and *M* = 2980.05, SD = 1545.51, respectively). This difference however was not significant with a two-tailed test, *t*(22) = 1.69, *p* = 0.11, Cohen’s *d* = 0.76. As can be seen in Supplementary Figure SA1, RT for the first item increased with set size only in the group of participants who selected targets to enhance memory performance.

##### Reaction Time for the Last Target in the Sequence (Averaged Across Set Sizes 2–7)

RTs for the last target were longer in the group of participants who followed the competition instructions relative to participants who did not follow these instructions (*M* = 3606.09, SD = 1688.14 and *M* = 2214.71, SD = 574.48, respectively). The difference was significant *t*(22) = 2.25, *p* < 0.05, Cohen’s *d* = 1.10.

##### Path Length (Averaged Across Set Sizes 2–7)

The average path length was significantly longer in the group of participants who followed the competition instructions (*M* = 4.54^0^, SD = 0.48) compared to the group of participants who reported selecting memory representations to enhance performance (*M* = 3.97^0^, SD = 0.33), *t*(22) = 3.08, *p* < 0.01, Cohen’s *d* = 1.38.

##### Path Crossings (Averaged Across Set Sizes 4–7)

The number of path crossings was similar in the two groups of participants (*M* = 1.43, SD = 0.13, and *M* = 1.49, SD = 0.17, for the participants who followed the competition instructions and those who did not follow it, respectively), *t*(22) = −1.00, *p* = 0.33, Cohen’s *d* = −0.40.

##### Configuration Size (Averaged Across Set Sizes 2–7)

The size of the spatial configuration was significantly larger in the group of participants who followed the competition instructions (*M* = 2.99^0^, SD = 0.18) relative to the group of participants who did not follow these instructions (*M* = 2.71^0^, SD = 0.16), *t*(22) = 3.66, *p* < 0.001, Cohen’s *d* = 1.64.

##### Accuracy

Accuracy was evaluated by a mixed model ANOVA with probe type as a within participant factor and group as a between-participant factor. Accuracy in the two groups was similar and high overall. The results of the ANOVA yielded a non-significant main effect of group *F*(1,22) < 1, probe *F*(1,22) = 1.36, *p* = 0.26, $\eta_{p}^{2}$ = 0.058, and a non-significant interaction between them *F*(1,22) < 1.

### Discussion

The main question addressed in Experiment 2 was whether participants would construct less structured spatial sequences when asked to construct spatial sequences that would disrupt memory performance. Indeed, relative to the representations in the SI condition of Experiment 1, the memory representations constructed in the competition condition consisted of fewer linear shapes, were overall larger, and had a longer average path length. Participants’ verbal reports focused exclusively on non-spatial strategies. The results of Experiment 2 further suggest that the spatial structure that participants imposed on the representations they constructed in the SI condition was intended to enhance memory rather than ease selection processes during encoding, as this structure was abolished when participants were asked to construct representations that would disrupt memory performance. Thus, participants in this experiment also had accesses to the metacognitive knowledge on the role of space in VWM. Although this knowledge was implicit (based on verbal reports), participants exerted control over the spatial strategies, by flexibly manipulating the spatial characteristics of the memory displays to disrupt memory performance.

The number of path crossings was the same in the competition and the SI conditions of Experiment 1, suggesting that participants identified proximity as the major factor that influenced memory performance. Alternatively, it is possible that participants have no accesses to the metacognitive knowledge on the impact of path crossings on performance. This explanation is less likely since participants in a previous spatial SI WM task did increase the number of path crossings in a competition condition compared to a SI condition (Magen and Emmanouil, 2018).

While less structured than in the SI condition, the memory representations in the competition condition were more structured than in the random non-SI condition, which suggests that the initial tendency is to construct structured spatial sequences that is manipulated and disrupted in the competition condition. Note that when the eight participants who did not follow the competition instructions were removed from the analysis, the difference between the competition and non-SI conditions remained significant in path length and the number of path crossings, but the overall size of spatial configurations was similar in these two encoding conditions.

Encoding RT for the last target in the sequence was longer in the competition condition relative to the SI and non-SI conditions. This finding confirms that participants in the competition condition constructed memory representations that they perceived to be more challenging. Nevertheless, accuracy was similar in the competition and SI conditions demonstrating that participants in Experiment 2 also adjusted encoding RT to reach almost ceiling performance.

Finally, 8 of the 24 participants in Experiment 2 did not follow the competition instructions. Comparing the results of the participants who attempted to enhance memory performance to those who attempted to disrupt memory performance showed clear differences in the spatial characteristics of the memory displays they constructed. This dissociation within the results of Experiment 2 provided additional support for the overall results of this study, and especially for the direct association participants (implicitly) made between spatial structure and the ease or difficulty of memory performance.

## General Discussion

Various situations in everyday life require individuals to shape the content of their memory representations. We have recently began to explore this aspect of memory we termed SI memory. In the current study, we focused on the spatial structure of SI VWM representations in a memory task in which space was task irrelevant. The results of two experiments demonstrated that when asked to enhance memory performance, participants planned and constructed spatially structured memory representations that relative to random provided representations, were overall smaller, consisted of more linear shapes and the spatial sequence path was on average shorter and consisted of fewer path crossings. These spatial structures were mostly compatible with the results of our previous studies on spatial SI WM (Magen and Emmanouil, 2018; Milchgrub and Magen, 2018) and with the literature on structured provided spatial WM (e.g., Parmentier et al., 2005; Guerard et al., 2009). When asked to disrupt memory performance, participants constructed less spatially structured representations, which relative to the SI representations, were larger, with longer paths, and with fewer linear shapes. The number of path crossings, in contrast, was similar in the two experiments, which suggests that participants considered the size and shape of the overall spatial configuration as the important characteristics that influence memory performance in SI VWM. Because encoding was self-paced and participants were presented with a single central probe during retrieval, we speculate that the construction of the spatially structured configurations was intended to benefit maintenance.

Participants provided verbal reports on the strategies they used to construct the memory representations. Participants in the SI condition indicated that they most frequently selected targets that could be grouped by semantic categories or visual features. These very same non-spatial grouping strategies were abolished (i.e., used in the opposite way) in the competition condition when participants were asked to disrupt memory performance. The spatial aspects of the constructed memory representations were largely absent from participants’ verbal reports, although spatial structure was clearly imposed on the memory representations. Moreover, the spatial structure likely interfered with the implementation of the non-spatial strategies, since it constrained the choices that could be made based on the non-spatial grouping cues.

An interesting aspect of the present study is the finding that participants invested time and resources in the construction of spatially structured representations (as demonstrated by encoding RT), but were largely unaware of it. While we cannot isolate encoding RT related to the selection of the visual and the spatial characteristics of the constructed memory representations, our previous studies have shown that the construction of structured spatial representations by themselves was time consuming (Magen and Berger-Mandelbaum, 2018; Magen and Emmanouil, 2018). It would be interesting in future studies to tease apart the construction of the visual and spatial characteristics of the memory representations. These processes may operate independently as behavioral and neural evidence on provided VWM suggest that visual targets and the spatial configurations in which they are embedded in WM are dissociable (Ackerman and Courtney, 2012; Xie and Zhang, 2017).

In this and in our previous studies, we claim that SI WM representations are based on metacognitive knowledge on the basic structure of WM representations. As far as we know, the ability to apply this metacognitive knowledge in the construction of one’s own memory representations remains an unexplored topic. The results of the current study suggest that this knowledge can guide strategy selection in a controlled way that has a profound influence on behavior, but nevertheless remain implicit. These results are consistent with findings and models from the research literature on metacognition, which had suggested that implicit metacognitive knowledge can guide strategy selection and implementation, and that controlled metacognitive processes can be guided by different degrees of metacognitive awareness (Cary and Reder, 2002). The results of Experiment 2 are even more intriguing in this regard, as they show that even the more sophisticated strategic controlled processes in the competition condition (i.e., manipulating structure in the opposite way) remain implicit.

Thus, the implementation of the spatial strategies in the construction of SI memory representations and their absence from the participants’ verbal reports revealed different degrees of metacognitive awareness of this knowledge. We are unaware of previous studies that can illuminate this aspect of the results. However, some insights can be gained from studies in perception, which have shown dissociations in the estimated strength of different Gestalt organization cues when objective and subjective measures were used (Schmidt and Schmidt, 2013; Montoro et al., 2017). For instance, Schmidt and Schmidt (2013) showed that grouping by shape produced stronger effects on (objective) behavior than grouping by brightness, although both cues were judged to be equal in strength based on subjective ratings. The authors suggested that although the objective and subjective measures relied on the same visual input, the strength of each grouping cue was represented differently when it served the objective and subjective tasks. Unlike the studies just described, the tasks in this study were all self-initiated and subjective in nature, and organization was constructed by individuals rather than passively perceived. That said, the idea that organization in visual representations can be represented distinctly along different levels of the cognitive system is relevant and would be an interesting topic for future studies.

The memory representations constructed in the current study were spatially structured, consistent with our previous spatial SI WM studies (Magen and Emmanouil, 2018; Milchgrub and Magen, 2018). Nevertheless, several dissociations that were observed between this and the previous studies could point to fundamental differences between spatial and visual SI WM. First, the overall shape of the spatial structures constructed in the current study was less organized than in the previous spatial SI WM studies, consisting of fewer linear shapes. Furthermore, in our previous spatial SI WM tasks, we observed a consistent bias to initiate the spatial sequence at the top left side, which did not occur in the current study. Thus, while the spatial structure was shown to be quite robust in this SI VWM task, participants probably first selected visual targets that matched their non-spatial strategies and then built around them a structured spatial configuration that matched the non-spatial rule as best as possible.

Further distinctions between spatial and visual SI WM were observed in the competition experiments. The number of path crossings was manipulated in the competition condition (i.e., increased relative to the SI condition) only in the spatial SI WM tasks. Thus, while in the tasks used in the current study participants minimized the number of path crossings to enhance performance, and therefore on some level acknowledged its potential impact on memory performance, this aspect of the spatial configuration was overlooked when participants attempted to disrupt memory performance. This dissociation between spatial and visual SI WM could suggest that the underlying mental models that guide the construction of the memory representations in these two types of tasks vary to some degree.

Differences in the underlying mental models or regularities in spatial and visual SI WM could also explain why RT for the first item in the sequence was longer in the competition condition relative to the SI condition in the current study, but not in our previous spatial SI WM studies (Magen and Emmanouil, 2018, see also Magen and Emmanouil, 2019). We speculate that structured spatial configurations may be governed by one set of regularities (e.g., path length, number of crossings) that could be implemented implicitly and could be easily abolished when required. Grouping of visual targets, on the other hand, could be based on several different types of regularities that participants employ explicitly, and that first need to be established before they are abolished. Furthermore, constructing representations to disrupt memory performance based on idiosyncratic regularities may require planning as well.

Finally, accuracy was almost at ceiling across all conditions. Nevertheless, the SI condition yielded significantly higher accuracy rates than the non-SI condition. This effect, however, was small and was restricted to the match trials, probably due to the overall high accuracy rates in this task. The advantage of the SI over the non-SI condition could be related to any number of factors, the structured nature of the SI representations, benefit from self-initiation, or both. In two previous studies on spatial SI WM, we found that accuracy was enhanced in the SI condition relative to the non-SI condition, even when they were matched in structure, demonstrating additive benefits of structure and self-initiation on performance (Magen and Emmanouil, 2018, 2019). Several processes may underlay this additional advantage of self-initiation. For instance, long-term memory performance is often enhanced for self-referenced or self-generated information (Slamecka and Graf, 1978; Cunningham et al., 2008). Furthermore, the control participants had over the memory displays in the SI condition may have increased their sense of agency, which has been shown to have beneficial effects on memory performance (e.g., Murty et al., 2015).

While the benefits in memory performance associated with self-initiation are important and should be explored in future studies, the focus of the present study was on the underlying structure of SI VWM representations. These representations reveal the manner by which individuals shape their world for short or long durations, behavior that is prevalent and critical for efficient everyday functioning, but is largely absent from the WM literature. More generally, only a small number of studies have examined the manner by which individuals organize their surroundings in other domains. For instance, Solman and Kingstone (2017) examined how individuals organize objects during online performance of a novel task the authors created. The results showed that participants adopted strategies that led them to organize their space in accordance with task demands. Several of the strategies were associated with task performance. The cognitive processes that underlay the self-initiated behavior of individuals as they shape their environment should gain more attention in the research literature. Within this vast topic, we have begun to outline the basic structure of SI WM representations, which capture individuals’ metacognitive knowledge regarding the structure of memory, the strengths and weaknesses of their cognitive system, and their adaptability to an ever-changing complex world.

## Data Availability Statement

The datasets generated for this study are available on request to the corresponding author.

## Ethics Statement

The studies involving human participants were reviewed and approved by The Hebrew University IRB. The patients/participants provided their written informed consent to participate in this study.

## Author Contributions

HM was responsible for the design and running of the study. HM and TE performed the analysis and wrote the manuscript.

### Conflict of Interest

The authors declare that the research was conducted in the absence of any commercial or financial relationships that could be construed as a potential conflict of interest.
