# Supplementary material for: Spatial Organization in Self-Initiated Visual Working Memory
Source: Front Psychol. 2019 Dec 13;10:2734. doi: 10.3389/fpsyg.2019.02734 (PMC6923243; doi:10.3389/fpsyg.2019.02734)
Supplement: Supplementary file 1 [file Data_Sheet_1.docx]

Competition-SI

Competition


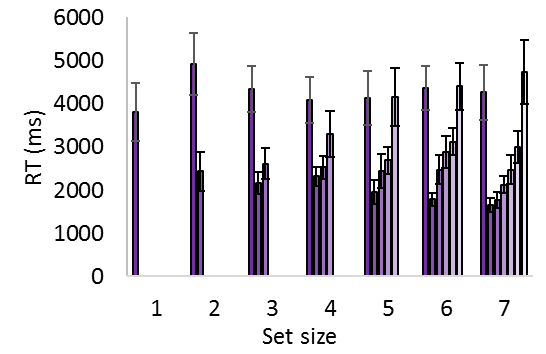

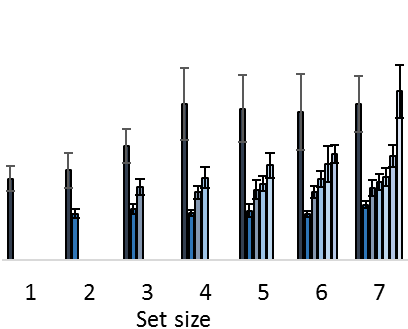


Competition SI serial position

4

1

2

3

5

6

7

Competition serial position

1

2

3

4

5

6

7

*Figure A1: Encoding RT as a function of serial position and set size in Experiment 2. Results are presented separately for participants who followed the competition instructions (‘Competition’), and participants who reported using strategies to enhance memory performance (‘Competition-SI). Except for the first target, encoding RT for each target was calculated with respect to the selection of the previous target in the sequence. Error bars represent standard error of the mean. SI – self-initiated.*

a.

b.

c.

Competition

SI

Competition-SI

*Figure A2: Characteristics of the spatial configurations in Experiment 2, as a function of set size. Results are presented separately for participants who followed the competition instructions (‘Competition’), and participants who reported using strategies to enhance memory performance (‘Competition-SI): a. the mean path length, b. the number of path crossings, c. configuration size. Error bars represent standard error of the mean. SI – self-initiated.*

Competition Match

Competition Non-Match

Competition Match

Competition Non-Match

Competition-SI Match

Competition-SI Non-Match

*Figure A3: Accuracy in Experiment 2, as a function of set size and probe condition (match or non-match). Results are presented separately for participants who followed the competition instructions (‘Competition’), and participants who reported using strategies to enhance memory performance (‘Competition-SI). Error bars represent standard error of the mean. SI – self-initiated.*
